# Pin Extracted from a Woman’s Throat

**Published:** 1889-07

**Authors:** Thomas Jay Pugh

**Affiliations:** Hearne, Texas


For Daniel's Texas Medical Journal.

### PIN EXTRACTED FROM A WOMAN'S THROAT.

BY THOS. JAY PUGH, HEARNE, TEXAS.

LAST FALL, latter part of November, a negro woman, age 19 years, was brought to my office from a neighboring Brazos bottom plantation, to have me extract a large pin from her throat. She was in the habit, as many other thoughtless people are, of making a repository of her mouth for her excess of pins. On this occasion she allowed a pin to be drawn down the throat beyond her control. As the pin descended it passed through the right tonsil, lacerating that organ considerably and finally anchored itself securely in the extreme base of the tongue, from which position I extracted it.

Now, there is nothing strange or new about this prelude. Yet I think the profession ought to have the benefit, if such it be, of my *mode* of procedure in relieving this unfortunate woman of a very embarassing condition. I attach my success to my *mode* and instrument. After ascertaining from the woman as well as I could the site of the intruding body, I had her to sit down on a chair and directed her to open her mouth wide and to protude her tongue forcibly out of her mouth, and not relax her effort. This effort brought the base of her tongue forward, and I, with my hand in her mouth up to the wrist joint, succeeded in getting the pin under the nail of my right index finger, and with difficulty I succeeded in withdrawing it from its lodgment. Had I resorted to forceps made for this purpose I would have failed. But the finger being an organ of touch, also a digito-crebro-telephone, I conceived it to be of superior value to any instrument usually used in such cases. The hand and digits form a wonderful instrument and I use it at all times for the removal of the adherent or detached placenta.

